# Liver elasticity measurement before and after biliary drainage in patients with obstructive jaundice: a prospective cohort studya prospective cohort study

**DOI:** 10.1186/s12876-016-0479-3

**Published:** 2016-07-08

**Authors:** Kimitoshi Kubo, Hiroshi Kawakami, Masaki Kuwatani, Mutsumi Nishida, Kazumichi Kawakubo, Shuhei Kawahata, Yoko Taya, Yoshimasa Kubota, Toraji Amano, Hiroki Shirato, Naoya Sakamoto

**Affiliations:** Department of Gastroenterology and Hepatology, Hokkaido University Hospital, Sapporo, Hokkaido Japan; Department of Gastroenterology and Hepatology, Center for Digestive Disease, University of Miyazaki, 5200 Kihara, Kiyotake-cho, Miyazaki City, 889-1692 Japan; Division of Endoscopy, Hokkaido University Hospital, Sapporo, Hokkaido Japan; Division of Laboratory and Transfusion Medicine, Hokkaido University Hospital, Sapporo, Hokkaido Japan; Department of Gastroenterology and Hepatology, Hokkaido University Graduate School of Medicine, Sapporo, Hokkaido Japan; Clinical Research and Medical Innovation Center, Hokkaido University Hospital, Sapporo, Hokkaido Japan; Department of Radiology, Hokkaido University Graduate School of Medicine, Sapporo, Hokkaido Japan

**Keywords:** Liver Elasticity, Obstructive jaundice, Biliary drainage, Transient elastography, Virtual Touch™ Quantification, Liver fibrosis, Hyaluronic acid, Procollagen-III-peptide

## Abstract

**Background:**

Obstructive jaundice has been reported to influence liver elasticity, independent of liver fibrosis. The aim of our prospective study was to evaluate the changes in liver elasticity, before and after biliary drainage, in patients with obstructive jaundice, and to evaluate the correlation between elasticity measures and serum markers of liver fibrosis.

**Methods:**

This is a prospective cohort study of 20 patients with obstructive jaundice. Liver elasticity was assessed by Transient Elastography (TE) and Virtual Touch™ Quantification (VTQ). Serum total bilirubin (T-Bil) level was measured before biliary drainage (Day 0), with measures repeated at 2 days (Day 2) and 7 days (Day 7) after biliary drainage. Serum levels of the following markers of liver fibrosis were also obtained on Day 0 and Day 7: hyaluronic acid (HA), procollagen-III-peptide (P-III-P).

**Results:**

T-Bil, TE, and VTQ for the left (VTQ-L) and right (VTQ-R) lobes of the liver were all elevated before biliary drainage, with respective levels, measured at Day 0, of 11.9 ± 1.5 mg/dl, 12.1 ± 0.9 kPa, 2.23 ± 0.10 m/s, and 1.85 ± 0.10 m/s. All values decreased on Day 7 after drainage: T-Bil, 4.7 ± 1.0 mg/dl (*P* < 0.001); TE, 7.6 ± 0.6 kPa (*P* < 0.001); VTQ-L, 1.53 ± 0.08 m/s (*P* < 0.001); and VTQ-R, 1.30 ± 0.05 m/s (*P* < 0.001). Similar changes were observed in serum markers of liver fibrosis. Liver elasticity measures correlated with serum levels of T-Bil, P-III-P, and HA (r = 0.35-0.67, *P* < 0.001).

**Conclusions:**

This study confirmed decreases in liver elasticity, measured by TE and VTQ, after biliary drainage. Measures of liver elasticity correlated to levels of T-Bil and serum markers of liver fibrosis. (UMIN ID: UMIN00001284313).

**Trial registration:**

Registration number: University Hospital Medical Information Network (UMIN) Clinical Trials Registry (UMIN ID: UMIN00001284313); Registration date: 2014-01-14.

## Background

The grade of liver fibrosis is a major indicator of the progression of chronic liver disease. Liver biopsy is considered to be an essential method [1] for conducting pathological evaluation of the fibrotic tissues. However, liver biopsy is associated with various complications, some of which can be life-threatening [2, 3]. As alternatives to liver biopsy, several non-invasive serum markers, such as hyaluronic acid (HA), procollagen-III-peptide (P-III-P), type IV collagen 7S (IV collagen), and two associated liver fibrosis scores, Forns’ Index and the Fibro Index, have been used to assess liver fibrosis [4–6]. Recently, several ultrasound-based methods have been developed to quantify liver elasticity for the assessment of liver fibrosis. The best known methods among these are Transient Elastography (TE) and Virtual Touch™ Quantification (VTQ) [7]. TE measures the propagation velocity of a single-cycle shear wave generated by a probe [8], with values expressed in units of pressure (kilopascal, kPa). In contrast, VTQ measures the propagation velocity of a transverse shear wave generated by a short-duration acoustic push pulse [9], with values expressed in units of velocity (meter per second, m/s). Compared to TE, VTQ offers the advantage of real-time elastography being integrated into a conventional ultrasound system. Furthermore, VTQ can be used to evaluate liver tissue over a wide range of depths and, therefore, provides a feasible method for the examination of patients with ascites for whom TE cannot be applied. Meta-analyses have provided evidence supporting both TE and VTQ as non-invasive methods for evaluating the stage of liver fibrosis, each providing high diagnostic accuracy to differentiate liver fibrosis and liver cirrhosis [10–12].

Obstructive jaundice has been known to be caused mainly by pancreaticobiliary diseases. Several studies have reported that biliary obstruction causes cholestatic injury to the liver, including hepatocellular necrosis and apoptosis, bile duct epithelial cell proliferation, and liver fibrosis [13–15]. As well, obstructive jaundice has been reported to be closely associated with changes in liver elasticity, independent of liver fibrosis [16–21]. However, the effects of obstructive jaundice on liver elasticity and the correlation of liver elasticity and non-invasive serum markers of liver fibrosis, such as HA and P-III-P, have not been examined and remain to be fully characterized.

The aim of our prospective study was to evaluate the changes in liver elasticity, measured before and after biliary drainage, in patients with obstructive jaundice using TE and VTQ, and to evaluate the correlation between liver elasticity measures, obtained with these two methods, and serum markers of liver fibrosis.

## Methods

### Patients

Participants for our prospective cohort study were patients with obstructive jaundice, admitted to Hokkaido University Hospital from July 2013 to January 2015. Prospective participants were screened on the following inclusion criteria: confirmed diagnosis of obstructive jaundice based on imaging examination; total serum bilirubin (T-Bil) level > 2 mg/dl, and clinical indication for biliary drainage. Patients with chronic liver disease, liver tumors, ascites, acute cholangitis, or contraindications to biliary drainage, were excluded. Written informed consent was obtained from all patients. This study was approved by our institutional review board (No. 013–0174) and was registered in the University Hospital Medical Information Network (UMIN) Clinical Trials Registry (UMIN ID: UMIN00001284313).

### Measurements of liver elasticity

A single experienced operator (K.K.) measured liver elasticity by TE, using a Fibroscan® (Echosens, Paris, France), and by VTQ, using ACUSON S2000® (Mochida SIEMENS Medical Systems, Tokyo, Japan). TE and VTQ measurements were completed at the same session for all patients. Values measured by TE were expressed in kPa, with a mean (range) value of 5.5 (1.5–12.7) kPA considered to be normal [22]. Values measured by VTQ were expressed in m/s, with a mean (range) value of 1.59 (0.76–3.43) m/s considered to be normal [23].

TE was measured for the right lobe of the liver, with access through the overlying intercostal space; and obtained under a condition of fasting. VTQ was measured at two sites, the right lobe of the liver (VTQ-R), accessed through the overlying intercostal space, and the left lobe of the liver (VTQ-L) which was also accessed through the overlying intercostal space but at a depth of 2–4 cm.

**Fig. 1:**
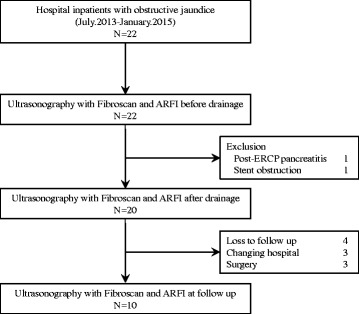
Flow chart of participant recruitment

Liver elasticity was measured in all patients before biliary drainage (Day 0), and at 2 days (Day 2) and 7 days (Day 7) post-biliary drainage. If possible, a fourth liver elasticity measurement was obtained during the follow-up period at the time when T-Bil level dropped to < 2 mg/dl (Fig. 1).

The results were considered to be reliable when a success rate of measurement of at least 60 % in 10 acquisition trials (i.e., ratio between validated and total measurements) was obtained. In addition, the median value was considered to be representative of TE and VTQ measurement only if the interquartile range (IQR) of all validated measurements was within 30 % of the median value.

### Serum markers of liver fibrosis and total bilirubin

Blood samples were obtained from all patients on the day when measurements of liver elasticity were performed. Levels of the following serum markers of liver fibrosis were examined: T-Bil level, HA, P-III-P, and IV collagen. T-Bil was examined at all time-points of measurement, while HA, P-III-P and IV collagen were examined at Days 0 and 7. The following serum liver fibrosis scores were calculated using previously published formulas:

1 $$ \begin{array}{l}\mathrm{Forns}'\ {\mathrm{Index}}^6 = 7.811-3.131. \ln\ \left(\mathrm{platelet}\ \left[{10}^9/\mathrm{L}\left]\left) + 0.781. \ln\ \right(\upgamma \hbox{-} \mathrm{G}\mathrm{T}\ \right[\mathrm{IU}/\mathrm{L}\right]\right)\hfill \\ {}\kern12em  + 3.467. \ln\ \left(\mathrm{age}\right)\ \hbox{-}\ 0.014\ \left(\mathrm{cholesterol}\left[\mathrm{mg}/\mathrm{dL}\right]\right)\hfill \end{array} $$

2 $$ \begin{array}{l}\mathrm{Fibro}\ {\mathrm{Index}}^4 = 1.738\hbox{-} 0.064\ \left(\mathrm{platelet}\ \left[\times {10}^4/{\mathrm{mm}}^3\right]\right)\kern1.5em  + 0.005\ \left(\mathrm{A}\mathrm{S}\mathrm{T}\ \left[\mathrm{IU}/\mathrm{L}\right]\right)\hfill \\ {}\kern15.5em  + 0.463\ \left(\upgamma \hbox{-} \mathrm{globulin}\ \left[\mathrm{g}/\mathrm{dl}\right]\right)\hfill \end{array} $$

3 $$ \begin{array}{l}\mathrm{A}\mathrm{spartate}\ \mathrm{A}\mathrm{minotransferase}\hbox{-} \mathrm{t}\mathrm{o}\hbox{-} \mathrm{platelet}\ \mathrm{Ratio}\ \mathrm{Index}\ {\left(\mathrm{APRI}\right)}^5=\hfill \\ {}\mathrm{A}\mathrm{S}\mathrm{T}\left[\mathrm{IU}/\mathrm{L}\right]\ \left(/\mathrm{U}\mathrm{L}\mathrm{N}\right) \times 100/\mathrm{platelet}\left[{10}^9/\mathrm{L}\right].\hfill \end{array} $$

### Biliary drainage

All patients underwent biliary drainage by endoscopic biliary stenting (EBS), endoscopic nasobiliary drainage (ENBD), or endoscopic ultrasonography-guided choledochoduodenostomy (EUS-CDS). Biliary drainage procedures were performed under conscious sedation, using fentanyl and midazolam by single experienced operator (H.K.). EBS and ENBD were performed using a duodenal endoscope (TJF-260 V, Olympus, Tokyo, Japan), a 0.025-inch guidewire (VisiGlide™, Olympus), and either a 7-Fr tube stent (Flexima, Boston Scientific Japan, Tokyo, Japan) or a 5-Fr or 6-Fr nasobiliary drainage tube (Sumitomo Bakelite, Tokyo, Japan or Olympus). EUS-CDS was performed using an echoendoscope (GF-UCT240-AL5, Olympus), a 19-gauge needle (Cook Japan, Tokyo, Japan), a guidewire, a 6Fr diathermic dilator (Cysto-Gastro-Set, Endo-flex, Voerde, Germany), and a partially covered self-expandable metallic stent (WallFlex™; 10 × 60 mm, Boston Scientific Japan).

### Sample size

In this study, we assessed whether liver elasticity was decreased after biliary drainage in patients with obstructive jaundice. Based on previously published clinical data [18], a 3.8 kPa change in TE values, before and after biliary drainage, was assumed. As no standard deviation data on TE measures of liver elasticity, measured pre- and post-biliary drainage, have been published, we assumed a moderate correlation (r = 0.5) between our scores and the mean change of 3.8 kPA. Based on this assumption, a sample size of 14 patients would provide 91 % power to detect a decrease in liver elasticity after biliary drainage, using a paired Student’s *t*-test, with a significance level of 0.05 (two-sided). Assuming there would be some dropouts of enrolled patients, our recruitment goal was set at 20 patients.

### Statistical analysis

Data were analyzed using JMP, version 10.0.2 (SAS Institute, Cary, NC, USA). Data were expressed as mean ± SE. Continuous variables were compared using paired Student’s *t*-tests. Correlations between parameters were determined by Pearson’s product–moment correlation coefficient. Differences were considered significant at *P* < 0.05.

## Results

### Patient characteristics

Twenty-two patients with obstructive jaundice associated to malignant tumors and meeting the clinical criteria for biliary drainage were enrolled into the study. Of these, 20 patients underwent liver elasticity measurement before and after biliary drainage, with the success rate of measurement and the median value of IQR all considered to be reliable by the validation criteria described in the previous section. For the other 2 patients, liver elasticity could not be accurately evaluated after biliary drainage due to post-ERCP pancreatitis and early stent obstruction. The data from these two patients was excluded from the analysis. Of these 20 patients, 4 patients were loss to follow-up, 3 patients were transferred to other hospitals, and 3 patients underwent surgery. Therefore, 10 patients underwent liver elasticity measurement in the follow-up period.

The relevant characteristics of the patients forming our study group are presented in Table 1 and summarized as follows: 12 males, mean age of 69.9 years, and mean body mass index of 22.0 kg/m^2^. All patients had carcinoma, originating from different primary sites: the pancreas in 10 patients, the bile duct in 7, the ampulla of Vater in 2, and the gallbladder in 1. The site of biliary obstruction was hilar in 7 patients and distal obstruction in 13 patients. Biliary drainage was performed by EBS in 7 patients, ENBD in 6 and EUS-CDS in 7. The mean duration of follow-up was 38.9 days (range, 8 to136 days).

**Table 1:** Patient characteristics

Male/Female	12/8
Age (mean, range)	69.9 (59–88)
Body mass index (kg/m^2^) (mean, range)	22.0 (16.1-33.6)
Underlying diseases	
Pancreatic carcinoma	10
Cholangiocarcinoma	7
Ampullary carcinoma	2
Gallbladder carcinoma	1
Endoscopic intervention	
EUS-CDS	7
EBS	7
ENBD	6
Observation period (mean, range)	38.9 (8–136)

*EUS-CDS* Endoscopic ultrasound (EUS)-guided choledochoduodenostomy, *EBS* Endoscopic biliary stenting, *ENBD* Endoscopic nasobiliary drainage

### Changes in T-Bil levels and liver elasticity

**Fig. 2:**
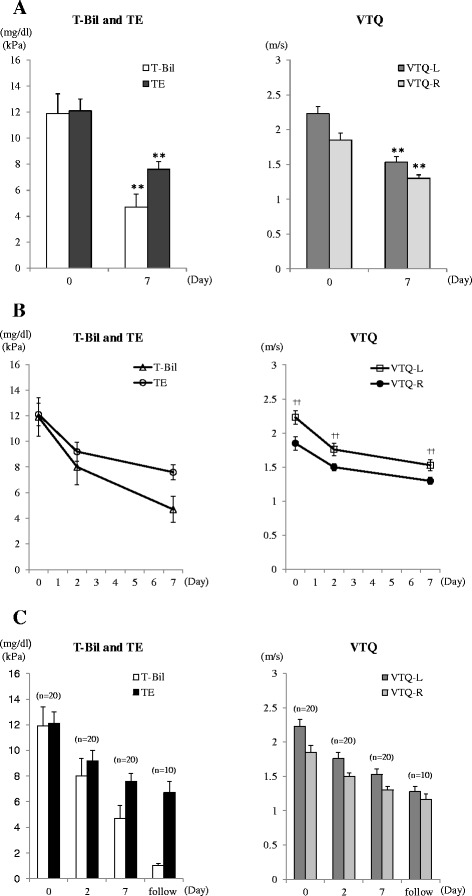
Changes in Serum Total Bilirubin (T-Bil) and Liver Elasticity. Liver elasticity was measured before and after biliary drainage. **a** Transient Elastography (TE) and T-Bil values were elevated before drainage and significantly decreased at Day 7 after biliary drainage (left). Similarly, Virtual touch quantification-Left lobe (VTQ-L) and Virtual touch quantification-Right lobe (VTQ-R) values were elevated before drainage and significantly decreased at Day 7 after biliary drainage (right). **b** T-Bil, TE, VTQ-L, and VTQ-R values decreased rapidly from Day 0 to Day 2 in comparison with from Day 2 to Day 7 after biliary drainage. VTQ-L value was significantly higher than VTQ-R value in each measurement date (right). **c** In 10 patients, who underwent further assessment after Day 7, TE, VTQ-L, and VTQ-R values further decreased. Data are shown as means ± SE; ** *P* < 0.01 versus Day0; ††*P* < 0.01 versus VTQ-R

T-Bil, TE, VTQ-L, and VTQ-*R* values were all elevated before biliary drainage, with values of: T-Bil, 11.9 ± 1.5 mg/dl, TE, 12.1 ± 0.9 kPa; VTQ-L, 2.23 ± 0.10 m/s; and VTQ-R 1.85 ± 0.10 m/s. All values decreased significantly at post-drainage Day 7, respectively, to values of: 4.7 ± 1.0 mg/dl (*P* < 0.001), 7.6 ± 0.6 kPa (*P* < 0.001), 1.53 ± 0.08 m/s (*P* < 0.001), and 1.30 ± 0.05 m/s (*P* < 0.001) (Table 2 and Fig. 2a). The rate of change in values was higher from Day 0 to Day 2, compared to the rate of change from Day 2 to Day 7 (Fig. 2b). For 10 patients, further measurements of liver elasticity were obtained during the follow-up period, with values further decreasing: TE, 6.7 ± 0.9 kPa; VTQ-L, 1.28 ± 0.07 m/s; and VTQ-R, 1.16 ± 0.08 m/s (Fig. 2c). In addition, the VTQ-*L* value was significantly higher than the VTQ-*R* value at each time-point of measurement (Fig. 2b).

**Table 2:** Liver elasticity, serum data, and serum markers, measured before and after biliary drainage

	Day 0 (*n* = 20)	Day 2 (*n* = 20)	Day 7 (*n* = 20)	> Day 7 (*n* = 10)	*P* ^*^
TE (kPa)	12.1 ± 0.9	9.2 ± 0.8	7.6 ± 0.6	6.7 ± 0.9	<0.001
VTQ-L (m/s)	2.23 ± 0.10	1.76 ± 0.09	1.53 ± 0.08	1.28 ± 0.07	<0.001
VTQ-R (m/s)	1.85 ± 0.10	1.50 ± 0.05	1.30 ± 0.05	1.16 ± 0.08	<0.001
T-Bil (mg/dl)	11.9 ± 1.5	8.0 ± 1.4	4.7 ± 1.0	1.0 ± 0.1	<0.001
AST (U/L)	209.8 ± 28.8	129.4 ± 23.0	66.8 ± 7.7	34.3 ± 6.6	<0.001
γ-GT (U/L)	1123.7 ± 166.2	839.0 ± 123.2	416.6 ± 63.7	202.4 ± 51.1	<0.001
ALP (U/L)	2,325.1 ± 217	1,987.9 ± 198.2	1,258.2 ± 126.5	682.8 ± 164.1	<0.001
Plt (×10^4^/μl)	25.9 ± 1.3	26.3 ± 1.5	29.2 ± 1.7	24.2 ± 1.7	NS
γ-globulin (g/dl)	1.20 ± 0.05		1.14 ± 0.06	1.09 ± 0.09	0.011
T-cho (mg/dl)	243.7 ± 17.5		186.5 ± 13.8	172.9 ± 12.4	<0.001
Hyaluronic acid (ng/ml)	324.2 ± 61.4		199.9 ± 41.6	118.0 ± 25.7	0.002
P-III-P (U/ml)	1.40 ± 0.10		1.14 ± 0.07	0.09 ± 0.08	0.004
IV collagen (ng/ml)	7.9 ± 0.9		7.2 ± 0.6	6.5 ± 0.8	NS
Forns' Index	6.98 ± 0.41		6.76 ± 0.42	6.56 ± 0.46	NS
Fibro Index	1.69 ± 0.18		0.73 ± 0.13	0.61 ± 0.25	<0.001
APRI	2.60 ± 0.40		0.79 ± 0.15	0.43 ± 0.11	<0.001

*TE* Transient Elastography, *VTQ-L* Virtual Touch™ quantification-Left lobe, *VTQ-R* Virtual Touch™ quantification-Right lobe, *T-Bil* total bilirubin, *AST* aspartate aminotransferase, *γ-GT* gamma-glutamyl transpeptidase, *ALP* alkaline phosphatase, *Plt* platelet, *T-cho* total cholesterol, *P-III-P* procollagen-III-peptide, IV collagen, type IV collagen 7S, *APRI* aspartate aminotransferase-to-platelet ratio index

All data are shown as means ± SE; *in comparison with Day 0 and Day 7

### Changes of serum markers of liver fibrosis

**Fig. 3:**
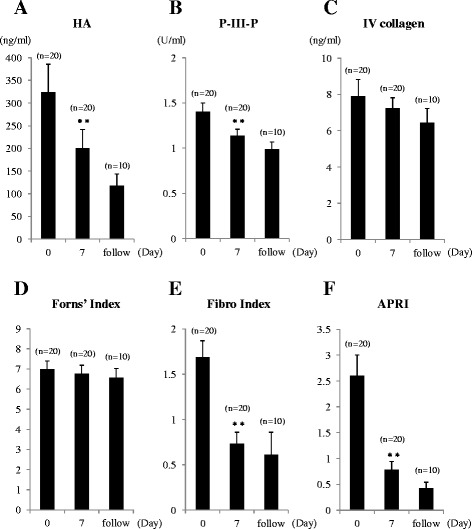
Changes in Serum Markers of Liver Fibrosis. **a** Hyaluronic acid (HA), **b** procollagen-III-peptide (P-III-P), **c** type IV collagen 7S (IV collagen), **d** Forns’ Index, **e** Fibro Index, and (**f**) APRI were measured, before and after biliary drainage. HA, P-III-P, Fibro Index, and APRI were elevated before drainage and decreased significantly at Day 7 after biliary drainage. IV collagen and Forns’ Index were elevated before drainage and tended to decrease at Day 7 after biliary drainage. In 10 patients who underwent assessment after Day 7, all the markers decreased. Data are shown as means ± SE; ** *P* < 0.01 versus Day 0

Values for serum markers and liver fibrosis scores were elevated in all patients before drainage: HA, 324.2 ± 61.4 ng/ml; P-III-P, 1.40 ± 0.10 U/ml; IV collagen, 7.9 ± 0.9 ng/ml; Forns’ Index, 6.98 ± 0.41; Fibro Index, 1.69 ± 0.18; and APRI, 2.60 ± 0.40. After biliary drainage, all values decreased at Day 7: HA, 199.9 ± 41.6 ng/ml (*P* = 0.002); P-III-P, 1.14 ± 0.07 U/ml (*P* = 0.004); IV collagen, 7.2 ± 0.6 ng/ml (*P* = NS); Forn's index, 6.76 ± 0.42 (*P* = NS); Fibro Index, 0.73 ± 0.13 (*P* < 0.001); and APRI, 0.79 ± 0.15 (*P* < 0.001) (Table 2 and Fig. 3a-f). Only after-drainage decreases in HA, P-III-P, FibroIndex, and APRI were significant. For the 10 patients evaluated over the follow-up period, HA, P-III-P, IV collagen, Forns’ Index, Fibro Index, and APRI values further decreased, respectively, to 118.0 ± 25.7 ng/ml, 0.09 ± 0.08 U/ml, 6.5 ± 0.8 ng/ml, 6.56 ± 0.46, 0.61 ± 0.25, and 0.43 ± 0.11 (Table 2 and Fig. 3a-f).

### Correlation between T-Bil, liver elasticity, and serum markers of liver fibrosis

**Fig. 4:**
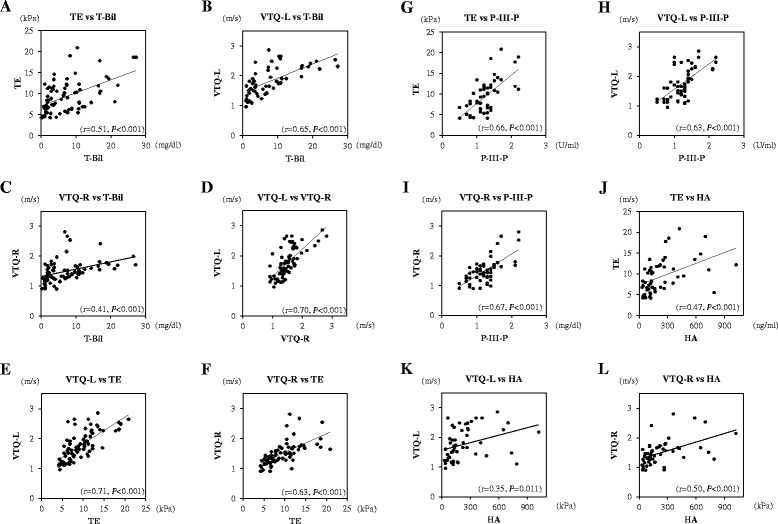
Correlations between T-Bil, Liver Elasticity, and Serum Markers of Liver Fibrosis. **a** TE and T-Bil (r = 0.51, *P* < 0.001), **b** VTQ-L and T-Bil (r = 0.65, *P* < 0.001), **c** VTQ-R and T-Bil (r = 0.41, *P* < 0.001), **d** VTQ-L and VTQ-R (r = 0.70, *P* < 0.001), **e** VTQ-L and TE (r = 0.71, *P* < 0.001), **f** VTQ-R and TE (r = 0.63, *P* < 0.001), **g** TE and P-III-P (r = 0.66, *P <* 0.001), **h** VTQ-L and P-III-P (r = 0.63, *P* < 0.001), **i** VTQ-R and P-III-P (r = 0.67, *P* < 0.001), **j** TE and HA (r = 0.47, *P* < 0.001), **k** VTQ-L and HA (r = 0.35, *P* < 0.001), **l** VTQ-R and HA (r = 0.50, *P* < 0.001)

The correlation between T-Bil, TE, VTQ-L, and VTQ-R values was tested. As shown in Fig. 4a-f, strong correlations were apparent between VTQ-L and VTQ-*R* values (r = 0.70, *P* < 0.001) and VTQ-L and TE values (r = 0.71, *P* < 0.001). In addition, moderate correlations were apparent between TE and T-Bil values (r = 0.51, *P* < 0.001), VTQ-L and T-Bil values (r = 0.65, *P* < 0.001), VTQ-R and T-Bil values (r = 0.41, *P* < 0.001), and VTQ-R and TE values (r = 0.63, *P* < 0.001). The correlation between liver elasticity and serum markers of liver fibrosis was evaluated for markers which significantly decreased after biliary drainage, namely P-III-P and HA. As shown in Fig. 4g-l, moderate correlations were apparent between TE and P-III-P values (r = 0.66, *P* < 0.001), VTQ-L and P-III-*P* values (r = 0.63, *P* < 0.001), VTQ-R and P-III-*P* values (r = 0.67, *P* < 0.001), TE and HA values (r = 0.47, *P* < 0.001), and VTQ-R and HA values (r = 0.50, *P* < 0.001). In addition, a weak correlation was apparent between VTQ-L and HA values (r = 0.35, *P* < 0.001). The correlations between serum markers and the Fibro Index and APRI were not evaluated as decreases in these ratios were considered to be influenced by the decrease in AST (Table 2).

### Correlation between alkaline phosphatase (ALP) and liver elasticity

The correlations between ALP, TE, VTQ-L, and VTQ-R were tested. Moderate correlation between VTQ-R and ALP values (r = 0.46, *P* < 0.01) was seen. In addition, weak correlation between VTQ-L and ALP values was also seen (r = 0.32, *P* < 0.01), while there was no correlation between TE and ALP values (r = 0.073, *P* = 0.54).

### Correlation among T-Bil, ALP, liver elasticity, and sites of biliary obstruction

In hilar biliary obstruction (*n* = 7), the correlation between T-Bil, ALP, TE, VTQ-R, and VTQ-L were tested. Moderate correlations were seen between TE and T-Bil values (r = 0.48, *P* < 0.01), VTQ-L and T-Bil values (r = 0.57, *P* < 0.01), while no correlation between VTQ-R and T-Bil values (r = 0.29, *P* = 0.13). In addition, weak correlation between VTQ-R and ALP values (r = 0.39, *P* < 0.04) was seen, while no correlations between TE and ALP values (r = 0.21, *P* = 0.29), and VTQ-L and ALP values (r = 0.19, *P* = 0.34) were seen.

Also in distal biliary obstruction (*n* = 13), the correlation between T-Bil, ALP, TE, VTQ-R, and VTQ-L were tested. Strong correlation was seen between VTQ-L and T-Bil values (r = 0.71, *P* < 0.01). Moderate correlations were seen between TE and T-Bil values (r = 0.45, P < 0.01), VTQ-R and T-Bil values (r = 0.48, *P* < 0.01), and VTQ-R and ALP values (r = 0.50, *P* < 0.01). Weak correlations were seen between TE and ALP values (r = 0.30, *P* < 0.047), VTQ-L and ALP values (r = 0.39, *P* < 0.01). In distal biliary obstruction, correlations were seen between all of T-Bil, ALP, TE, VTQ-R and VTQ-L.

## Discussion

The main findings of our study are that liver elasticity, measured by TE and VTQ, significantly decreased after biliary drainage together with serum markers of liver fibrosis (P-III-P and HA), and that liver elasticity correlated with levels of T-Bil, P-III-P, and HA. The rapid decrease in liver elasticity from Day 0 to Day 2 indicates that elevation in liver elasticity is influenced by elements other than pathological liver fibrosis. The profile of elevated liver elasticity before biliary drainage followed by a rapid decrease after biliary drainage has previously been reported in patients with obstructive jaundice [16–21]. The reported periods from biliary drainage to measurement of liver elasticity varied among these studies: 2–11 days [16], 1–2 days and 3.0 ± 9.31 weeks [17], 27–32 days [19], 5–45 days [20], and 3–12 days [21]. Recently, two studies have provided evidence of the usefulness of VTQ measures [16, 17]. However, one of these studies included elasticity measured for liver conditions other than obstructive jaundice, such as cholangitis [17], while the other was limited by its small sample size (10 patients) and variation in lapse of time between drainage and measurement of elasticity [16]. Our study excluded patients with chronic liver disease, liver tumors, ascites, and acute cholangitis, because these could also affect liver elasticity. Additionally, we measured liver elasticity within a very short lapse of time after biliary drainage (i.e., Day 2 and Day 7) to avoid bias of longer or varying observational period on measured values. More recently, it has been reported that operator-related and patient-related factors can produce significant variations in liver elasticity measurements made by TE, which limits the use of TE for longer-term monitoring of patients [24]. To avoid operator-related factors, liver elasticity was measured by a single experienced operator in our study. Accordingly, our results show more reliable measurement than those reported in previous studies.

Changes in the Fibro Index after biliary drainage reflect changes in AST and γ-globulin levels, while changes in APRI reflect changes in AST level. Therefore, we did not consider the Fibro Index and the APRI to directly reflect the stage of liver fibrosis. In addition, it has been reported that HA is markedly high in certain liver diseases, especially in patients with cirrhosis, and that P-III-P is strongly correlated with the histological degree of bridging in hepatocellular necrosis and liver fibrosis in periportal areas [25, 26]. In our study, HA and P-III-P were elevated before biliary drainage, with levels significantly decreasing after drainage. These changes in HA and P-III-P partially reflect amelioration of liver fibrosis in obstructive jaundice after drainage. The mechanisms by which high liver elasticity develops in obstructive jaundice, however, is still unknown. One study demonstrated that experimental blockage in bile flow resulted in visible swelling of the liver, accompanied by high liver elasticity [21].

VTQ is superior to TE in that VTQ can be used to separately measure elasticity in both the left and right lobes of the liver. A previous study reported VTQ to be an accurate, reliable, reproducible, and non-invasive method to assess liver fibrosis of both the left and right lobes [27]. The consistently lower VTQ values for the right, compared to the left, lobe of the liver reflects the influences of movement of body organs, such as heart, lungs, diaphragm, and stomach, against the left lobe of the liver [27].

A weak and moderate correlation was apparent between ALP and VTQ-L, and ALP and VTQ-R in this study, respectively. These results may be due to our setting of chronic biliary obstruction.

The limitations of our study need to be considered. This is a single center study, in which there is an inherent bias in patient selection due to the referral patterns to our institution. As well, measurements of liver elasticity were obtained by a non-blinded operator. The small sample size of this study and the constraints on patient selection may limit the ability to detect a higher correlation between T-Bil, TE, VTQ-L, and VTQ-R. A multi-center and larger cohort trial is needed to confirm our results. Moreover, due to its invasiveness, liver biopsy was not performed to rule out secondary liver fibrosis due to bile duct obstruction after biliary drainage. Additionally, in our study, correlation between liver elasticity and hepatic functional reserve, such as indocyanine green test and ^99m^Tc-GSA scintigraphy, was not evaluated. These associations should be investigated in future studies.

## Conclusions

Our study clearly showed a decrease in liver elasticity, evaluated by TE and VTQ, after biliary drainage. Changes in liver elasticity were correlated to changes in serum markers of liver fibrosis, P-III-P and HA specifically.

## Abbreviations

APRI, aspartate aminotransferase-to-platelet ratio index; AST, aspartate aminotransferase; EBS, endoscopic biliary stenting; ENBD, endoscopic nasobiliary drainage; EUS-CDS, endoscopic ultrasound-guided choledochoduodenostomy; HA, hyaluronic acid; IQR, interquartile range; IV collagen, type IV collagen 7S; P-III-P, procollagen-III-peptide; Plt, platelet; T-Bil, total bilirubin; T-cho, total cholesterol; TE, transient Elastography; VTQ, virtual touch™ quantification; VTQ-L, virtual touch™ quantification-left lobe; VTQ-R, virtual touch™ quantification-right lobe; γ-GT, gamma-glutamyl transpeptidase.
