# Erratum to: Liver elasticity measurement before and after biliary drainage in patients with obstructive jaundice: a prospective cohort study

**DOI:** 10.1186/s12876-016-0531-3

**Published:** 2016-09-20

**Authors:** Kimitoshi Kubo, Hiroshi Kawakami, Masaki Kuwatani, Mutsumi Nishida, Kazumichi Kawakubo, Shuhei Kawahata, Yoko Taya, Yoshimasa Kubota, Toraji Amano, Hiroki Shirato, Naoya Sakamoto

**Affiliations:** 1 Department of Gastroenterology and Hepatology, Hokkaido University Hospital, Sapporo, Hokkaido Japan; 2 Department of Gastroenterology and Hepatology, Center for Digestive Disease, University of Miyazaki, 5200 Kihara, , Kiyotake-cho Miyazaki City, 889-1692 Japan; 3 Division of Endoscopy, Hokkaido University Hospital, Sapporo, Hokkaido Japan; 4 Division of Laboratory and Transfusion Medicine, Hokkaido University Hospital, Sapporo, Hokkaido Japan; 5 Department of Gastroenterology and Hepatology, Hokkaido University Graduate School of Medicine, Sapporo, Hokkaido Japan; 6 Clinical Research and Medical Innovation Center, Hokkaido University Hospital, Sapporo, Hokkaido Japan; 7 Department of Radiology, Hokkaido University Graduate School of Medicine, Sapporo, Hokkaido Japan

Unfortunately, after publication of this article [1], it was noticed that the title was captured incorrectly during the production process. The words, “a prospective cohort study” were erroneously added twice. The corrected title can be seen above.
